# TNFα modulates protein degradation pathways in rheumatoid arthritis synovial fibroblasts

**DOI:** 10.1186/ar3778

**Published:** 2012-03-14

**Authors:** Alison M Connor, Nizar Mahomed, Rajiv Gandhi, Edward C Keystone, Stuart A Berger

**Affiliations:** 1 Arthritis and Immune Disorder Research Centre, University Health Network, Toronto Medical Discovery Tower, 10th Floor, Room 10-358, 101 College Street, Toronto, Ontario M5G 1L7, Canada; 2 Western Research Institute, University Health Network, 399 Bathurst Street, Toronto, Ontario M5T 3L9, Canada; 3 Division of Orthopaedic Surgery, University Health Network, 399 Bathurst Street, Toronto M5T 3L9, Ontario, Canada; 4 Department of Medicine, University of Toronto, 200 Elizabeth Street, Toronto, Ontario M5T 3L9, Canada; 5 The Rebecca MacDonald Centre for Arthritis and Autoimmune Disease, Mount Sinai Hospital, 60 Murray Street, Toronto, Ontario M5T 3L9, Canada; 6 Department of Immunology, University of Toronto, 1 King's College Circle, Toronto, Ontario M5S 1A8, Canada

## Abstract

**Introduction:**

Rheumatoid arthritis (RA) is a chronic inflammatory and destructive disease of the joint. The synovial lining consists of two main types of cells: synovial fibroblasts and macrophages. The macrophage-derived cytokine TNFα stimulates RA synovial fibroblasts to proliferate and produce growth factors, chemokines, proteinases and adhesion molecules, making them key players in the RA disease process. If proteins are not correctly folded, cellular stress occurs that can be relieved in part by increased degradation of the aberrant proteins by the proteasome or autophagy. We hypothesized that the activity of the protein degradation pathways would be increased in response to TNFα stimulation in RA synovial fibroblasts compared with control fibroblasts.

**Methods:**

Endoplasmic reticulum (ER) stress markers were examined in synovial fibroblasts by immunoblotting and PCR. Use of the autophagy and proteasome protein degradation pathways in response to TNFα stimulation was determined using a combination of experiments involving chemical inhibition of the autophagy or proteasome pathways followed by immunoblotting for the autophagy marker LC3, measurement of proteasome activity and long-lived protein degradation, and determination of cellular viability.

**Results:**

RA synovial fibroblasts are under acute ER stress, and the stress is increased in the presence of TNFα. Autophagy is the main pathway used to relieve the ER stress in unstimulated fibroblasts, and both autophagy and the proteasome are more active in RA synovial fibroblasts compared with control fibroblasts. In response to TNFα, the autophagy pathway but not the proteasome is consistently stimulated, yet there is an increased dependence on the proteasome for cell viability. If autophagy is blocked in the presence of TNFα, an increase in proteasome activity occurs in RA synovial fibroblasts but not in control cells.

**Conclusions:**

TNFα stimulation of synovial fibroblasts results in increased expression of ER stress markers. Survival of synovial fibroblasts is dependent on continuous removal of proteins by both the lysosome/autophagy and ubiquitin/proteasome protein degradation pathways. Both pathways are more active in RA synovial fibroblasts compared with control fibroblasts. These results may provide a better understanding of the mechanism of TNFα on prolonging the survival of synovial fibroblasts in RA tissue.

## Introduction

Rheumatoid arthritis (RA) is a chronic disease characterized by inflammation of the synovial membrane lining the joints, leading to cartilage and joint destruction. The synovial lining is composed of macrophages, B cells, T cells and synovial fibroblasts. The synovial fibroblasts are greatly expanded in number via a process driven by cytokines, especially the macrophage-derived TNFα. The cytokine TNFα stimulates proliferation and the production of additional cytokines, proteases and adhesion molecules. The underlying disease mechanism of RA is not understood, although resistance of the synovial fibroblasts to TNFα-induced apoptosis has been recognized as an important factor [1].

Fibroblasts are highly metabolic cells, synthesizing components of the extracellular matrix as well as proteases capable of degrading the extracellular matrix. For example, it is estimated that each cell can synthesize up to 3.5 million procollagen molecules per day [2]. Newly synthesized proteins that are destined for secretion or insertion into the plasma membrane are translocated into the endoplasmic reticulum (ER), where they undergo folding, post-translational modifications and examination by a quality control mechanism. Misfolded proteins are ubiquitinated and retrotranslocated by chaperone proteins to the cytosol, where they are degraded by cytosolic proteasomes. This process is known as endoplasmic reticulum-associated degradation [3]. ER stress occurs when levels of misfolded proteins exceed the capacity of the protein folding and endoplasmic reticulum-associated degradation systems, or when there is a change in the calcium regulation or oxidative stress in the ER. In this case, the unfolded protein response (UPR) is triggered. There are three pathways involved in the initiation of the UPR: protein kinase-like endoplasmic reticulum kinase (PERK), the inositol-requiring transmembrane kinase and endonuclease 1α (IRE1α), and the activation of transcription factor 6 (ATF6). The UPR involves phosphorylation of the translation initiation factor eukaryotic initiation factor 2α (eIF2α), resulting in inhibition of most new protein synthesis, activation of the transcription factor XBP-1 and increased expression of ER chaperone proteins such as Bip/GRP78. These changes enable the cell to repair misfolded proteins and upregulate the proteasomal degradation system to eliminate aberrant proteins [4].

If the UPR cannot relieve the ER stress, a lysosome-dependent degradation process known as autophagy may be activated [5]. Although autophagy is best known for its role in generating amino acids and energy required for cell survival during periods of nutrient deprivation and hypoxia, it has also been implicated as a pathway for the elimination of aberrant proteins. Macroautophagy is generally considered to be the most important pathway through which aberrant proteins are brought to the lysosome and it is characterized by sequestration of cytosolic regions in double-membrane autophagic vesicles (autophagosomes) that are then fused to and degraded by the lysosome and vacuole systems [6]. Microautophagy involves the direct uptake of cytoplasmic compounds by lysosomes. Chaperone-mediated autophagy utilizes chaperone proteins to transport proteins bearing a targeting motif to lysosomes, where they are translocated across the lysosomal membrane and degraded.

Excessive ER stress overwhelms the protein degradation systems and the cell ultimately undergoes apoptosis through the induction of pro-apoptotic transcription factors such as ATF4 and CHOP [7].

RA synovial fibroblasts have been demonstrated to be relatively resistant to ER stress-induced apoptosis when compared with osteoarthritis fibroblasts, HeLa or HEK293 cells, and this has been attributed to hyper-endoplasmic reticulum-associated degradation and the expression of synoviolin, the TNFα-inducible E3 ubiquitin ligase [8]. More recently, it has been shown that induction of autophagy also protects RA synovial fibroblasts from ER stress [9]. Interestingly, several studies suggest that the ER stress pathway and autophagy influence each other. However, the relative roles of autophagy and proteasome-mediated protein degradation in RA synovial fibroblasts, particularly under the influence of TNFα, have not been addressed.

In this study, we investigated the influence of TNFα on the relative role of these degradation pathways in RA synovial fibroblasts. Our findings suggest that fibroblasts use both the proteasome and lysosome/autophagy pathways to clear excess protein and promote survival. TNFα induces a partial ER stress response in synovial fibroblasts and sensitizes them to proteasome inhibition. TNFα consistently stimulates autophagy but not the proteasome. When either protein degradation pathway is inhibited, however, RA synovial fibroblasts initially compensate for the inhibition by upregulating the alternate protein degradation pathway.

## Materials and methods

### Synovial tissue

The ethics review committee at the University Health Network approved the protocol for patient consent and use of tissues. Synovial tissue from consented patients was obtained at the time of arthroplasty. Synovial fibroblasts were isolated from synovial tissue and maintained in Opti-MEM (Life Technologies Inc. Carlsbad, CA, USA) as described elsewhere [10].

### Cell culture

Adult dermal fibroblasts and skin lines were purchased from ATCC (American Type Culture Collection, Manassas, VA, USA) and maintained as described for the synovial fibroblasts.

### Chemicals

Unless otherwise indicated, all chemicals were from Sigma-Aldrich (Oakville, ON, Canada).

### Immunoblotting

Cells were plated at 1×10^5 ^cells per well in six-well culture dishes. Forty-eight hours later, additives (10 ng/ml TNFα (R&D Systems Inc., Minneapolis, MN, USA), 12.5 μM chloroquine, 4 mM 3-methyladenine (3-MA), 2 μg/ml tunicamycin, 0.5 μM MG132 or 0.5 μM epoxomicin) were included in the culture as indicated for a further 72 hours. Chloroquine is a weak base that accumulates inside lysosomes, preventing lysosomal acidification. This results in the inactivation of lysosomal hydrolases [11] and inhibits the late-stage step in autophagy that involves the fusion of autophagosomes with lysosomes [12]. In contrast, 3-MA inhibits class III phosphatidylinositol 3-OH kinase that is required for autophagosome formation, an early stage in autophagy [13]. Tunicamycin blocks the synthesis of all N-linked glycoproteins [14] and is used to induce ER stress. MG132 is a peptide aldehyde proteasome inhibitor, while epoxomicin is a natural proteasome inhibitor [15]. The concentrations of the inhibitors we used were based on those reported in the literature and preliminary titration experiments.

At the time of harvest, the plates were placed on ice, media were removed, plates were rinsed twice with PBS and whole cell lysates were prepared by adding SDS-PAGE lysis buffer (1.1% SDS, 11% glycerol, 88 mM Tris-HCl, pH 6.8) directly to the wells for 10 minutes. Lysate was collected and boiled for 5 minutes prior to shearing the DNA with a 22-gauge needle. A one-tenth volume of β-mercaptoethanol containing bromophenol blue loading dye was then added to the lysates such that the final concentration of loading dye was 0.01%. Proteins were separated by 12% SDS-PAGE, transferred to Immobilon-P membranes (Millipore Corp., Bedford, MA, USA) and probed for ser51-phosphorylated eIF2α, eIF2, microtube-associated light chain 3 (LC3) (Cell Signaling Technology, Beverly, MA, USA), ATF6 (Imgenex, San Diego, CA, USA), p62 and Bip/GRP78 (BD Biosciences Pharmingen, Mississauga, ON, Canada) in Tris-buffered saline/5% BSA. Blots were stripped between probings with Re-Blot-Plus (Chemicon International, Inc., Temecula, CA, USA). Loading was corrected by probing the blots for tubulin (NeoMarkers Inc., Fremont, CA, USA). In order to detect monoubiquitinated and polyubiquitinated proteins using clone FK2 (Enzo Life Sciences Inc., Farmingdale, CA, USA), it was necessary to decrease the amount of lysate loaded on the gel to 3 μg and decrease the concentration of BSA in the hybridization buffer to 1%. Blots were scanned and band intensities were determined by ImageJ software (National Institutes of Health, Bethesda, MD, USA).

### PCR analysis

Cells (1×10^5 ^cells per well) were stimulated in six-well culture dishes as indicated in the figure legends. Media were removed and RNA was prepared with the RNeasy Mini kit (Qiagen Inc., Mississauga, ON, Canada) according to the manufacturer's directions. cDNA was prepared from 50 ng RNA using the Sensiscript RT kit (Qiagen Inc.). PCR was performed with HotStarTaq DNA polymerase (Qiagen Inc.). The primers used are presented in Table 1.

**Table 1:** Primers used for PCR analysis

Actin	Forward	5'-ATGGCCACGGCTGCTTCCAGC-3'
	Reverse	5'-CATGGTGGTGCCGCCAGACAG-3'
CHOP	Forward	5'-CTGAGTCATTGCCTTTCTCCTTC-3'
	Reverse	5'-CTCTGACTGGAATCTGGAGAG-3'
Xbp-1	Forward	5'-TTACGAGAGAAAACTCATGGC-3'
	Reverse	5'-GGGTCCAAGTTGTCCAGAATGC-3'
Edem1	Forward	5'-CTGGCACGGGGCATGTTCGT-3'
	Reverse	5'-CAAAAGCAGGGAGGAGCCGCA-3'

Amplification conditions were 95°C for 15 minutes followed by: actin, 27 cycles of 92°C for 1 minute, 60°C for 1 minute, and 72°C for 1 minute; and Xbp-1, Edem1 and CHOP, 31 cycles of 92°C for 1 minute, 58°C for 1 minute, and 72°C for 1 minute, and a final extension step at 72°C for 10 minutes. The Actin, Edem1 and CHOP PCR products were resolved on a 1% agarose/Tris-acetate-EDTA gel. The endoribonuclease activity of activated IRE1 cleaves a 26-nucleotide Pst1-containing intron from Xbp1 mRNA. Xbp1 PCR products were therefore cleaved with Pst1 and resolved on 2% agarose/Tris-acetate-EDTA gels as an indirect indicator of IRE1 activation. Cleaved Xbp1is an active transcription factor, implicated in the expression of Edem1. Expression of Edem1 served as further evidence for IRE1/Xbp1 activation. Quantification was performed with ImageJ software. Relative amounts of Xbp-1 and CHOP were calculated from normalized actin.

### Microscopy

Cells were grown and stimulated on eight-well chamber slides (Lab-Tek, Nalge Nunc International, Naperville, IL, USA). The slides were rinsed with PBS and then fixed with 2% paraformaldehyde for 15 minutes. Slides were then rinsed three times with PBS prior to the addition of 0.1% saponin. Slides were rinsed an additional three times with PBS and then blocked with blocking buffer (PBS containing 5% BSA, 0.3% Triton-X100) for 1 hour. A 1/500 dilution of primary LC3 antibody (Cell Signaling Technology) was prepared in antibody dilution buffer (PBS containing 1% BSA, 0.3% Triton-X100), added to the slide and left overnight at 4°C. Slides were rinsed with PBS and then incubated for 1 hour at room temperature with a 1/500 dilution of goat-α-rabbit secondary antibody conjugated to Alexa-Fluor 488. A 1/1,000 dilution of DAPI (Calbiochem, Novabiochem Corp, San Diego, CA, USA) was added for the final 5 minutes in order to visualize the nucleus. The slides were rinsed three times in PBS and treated with the SlowFade Antifade Kit (Molecular Probes, Life Technologies Inc. Carlsbad, CA, USA) according to the manufacturer's specifications.

### Long-lived protein degradation assay

The long-lived protein degradation assay was modified from published procedures [16,17]. Cells were plated at 40,000 cells per well on 12-well plates. Long-lived proteins were labeled by removing the media, rinsing the cells once with PBS and culturing in the presence of 1 ml leucine-free media containing 10% serumand 5 μCi/ml [^3^H]-leucine for 48 hours. After the labeling media was removed, unincorporated radioisotopes and degraded amino acids were removed by rinsing the plate three times with PBS. Short-lived proteins were depleted by culturing the labeled cells with 1 ml Opti-MEM containing 4% serum and 2 mM cold leucine for 24 hours. The chase medium was removed, cells were rinsed once with PBS and additives were added in Opti-MEM/4% serum. Aliquots of the medium were removed at 24 hours, BSA was added to 3 mg/ml final concentration and trichloroacetic acid (TCA) was added to 10% final concentration. Proteins were precipitated by incubating at 4°C for 1 hour. Precipitates were recovered by centrifugation at 15,000×*g *for 5 minutes at 4°C. Supernatants were collected and pellets were washed with cold 20% TCA. The washes were combined with the supernatants and this fraction represented small cleaved protein fragments. PBS containing 0.5% Triton-X100 was added to the cells on the plate in order to recover counts associated with the cells. After a 1-hour incubation, the lysate was removed and the wells were rinsed with PBS containing 0.5% Triton-X100. TCA precipitations were then performed on the protein lysates as described above. Finally, SDS-PAGE lysis buffer was added to the wells to collect any remaining counts. TCA precipitates were air-dried and then resuspended in 0.1 N NaOH prior to counting. Aliquots of all fractions were counted with a scintillation counter. Proteolysis was determined as the ratio of non-TCA precipitable counts to the total counts in each well.

### Cell-based proteasome activity assay

Cells were plated in black 96-well plates (Packard Biosciences, Meriden, CT, USA) at 5,000 cells per well. They were cultured in Opti-MEM containing 4% serum with chloroquine 1 hour prior to adding 10 ng/ml TNFα. After 22 hours, the media were removed and cells were cultured in EBSS buffer (120 mM NaCl, 5.4 mM KCl, 0.81 mM MgSO_4_, 1 mM NaH_2_PO_4_, 5.5 mM D-glucose, 0.2 mM CaCl_2_, 25 mM HEPES) containing the indicated additives. For the 2-hour proteasome assays, there was no prior treatment with the additives in Opti-MEM. Where indicated, epoxomicin - an inhibitor of the chymotrypsin-like activity of the 20S proteasome [18] - and calpain inhibitor X1 were added for 1 hour prior to the addition of TNFα. Proteolytic activity was determined by the addition of the synthetic peptide Suc-Leu-Leu-Val-Tyr-AMC (LLVY) prepared in EBSS and digitonin directly to the wells such that the final concentration of substrate was 50 μM and that of digitonin was 13.3 μg/ml. Fluorescence measurements were performed every 5 minutes over 45 minutes on a SpectraMax M5 microplate reader (Molecular Devices, Sunnyvale, CA, USA). The excitation wavelength was 355 nm and the emission wavelength was 465 nm. Proteasome activity was calculated from slopes of the change in fluorescence over the change in time. In all cases, the slope of non-induced cells was set at 100%.

### XTT assay

Fibroblasts were plated at 3,000 cells per well in 96-well plates. Quadruplicate wells were treated for 72 hours with additives as indicated in the figure legends, and their viability was determined with an XTT assay as described elsewhere [10].

### Statistical analysis

Results are plotted as the mean with the standard error of the mean. Significant differences between groups were determined using the Student *t *test. *P *< 0.05 was considered significant.

## Results

### TNFα stimulates the acute endoplasmic reticulum stress response in RA synovial fibroblasts

Since it has been reported that RA synovial fibroblasts are relatively resistant to ER stress [8,9] and TNFα-induced reactive oxygen species accumulation has been shown to stimulate the UPR in murine fibrosarcoma L929 cells [19], we asked whether TNFα modulated the UPR of RA synovial fibroblasts. It has been suggested that the cell senses the severity of ER stress by integration of signals from the three different pathways in the UPR. The molecules that sense ER stress are PERK, IRE1 and ATF6. In the nonstressed cell, these molecules are maintained in an inactive state by association with the ER chaperone protein Bip. When ER stress occurs, Bip preferentially binds to aberrantly folded proteins that accumulate in the ER, thereby freeing the sensors to activate their signaling pathways.

**Figure 1:**
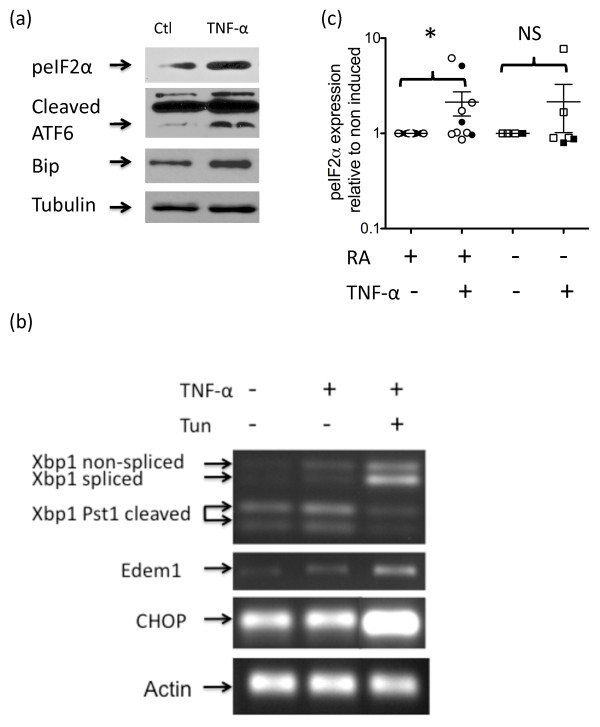
**Rheumatoid arthritis synovial fibroblasts exhibit acute endoplasmic reticulum stress**. **(a) **Synovial fibroblasts from patients with rheumatoid arthritis (RA) were stimulated or not with 10 ng/ml TNFα for 72 hours prior to protein isolation. After fractionation of the lysates by SDS-PAGE, they were immunoblotted and probed for expression of phosphorylated eukaryotic initiation factor 2α (peIF2α), activation of transcription factor 6 (ATF6), Bip or tubulin. Results are representative of at least three different RA lines. **(b) **Cells were cultured for 72 hours with or without the addition of 10 ng/ml TNFα or 2 μg/ml tunicamycin. RNA was isolated, reverse-transcribed and amplified for Xbp1, Edem1, CHOP or actin. Xbp1 amplification products were subsequently subjected to Pst1 cleavage before analysis on agarose gels. Results shown are representative of four different RA lines. **(c) **Fibroblasts were treated with 10 ng/ml TNFα or were left untreated for 24 or 72 hours prior to the isolation of cellular lysates. Following SDS-PAGE and immunoblotting, band intensities of peIF2α were determined and normalized to tubulin levels to compensate for loading differences. The normalized peIF2α levels of TNFα-stimulated cells were then compared with the nonstimulated control. Filled circles, RA synovial fibroblasts at 24 hours; open circles, RA synovial fibroblasts at 72 hours; filled squares, control fibroblasts at 24 hours; open squares, control fibroblasts at 72 hours. Significant differences in TNFα-stimulated cultures compared with non-induced cultures: *P < 0.05. NS not significant.

We evaluated the expression of signature UPR markers within each of the three initiation pathways in synovial fibroblasts derived from patients with RA. These included eIF2α that is phosphorylated by active PERK, Xbp1 mRNA that has an intron removed by active IRE1α, and ATF6 protein that is proteolytically processed to its active form in the golgi in response to ER stress. Additionally, we examined expression of the ER chaperone protein Bip/GRP78 and the proapoptotic transcription factor CHOP. We observed that expression of phosphorylated eIF2α, the active/cleaved forms of ATF6 proteins and Bip protein were all increased in RA synovial fibroblasts chronically stimulated by TNFα compared with nonstimulated cells (Figure 1a). Although nonstimulated or TNFα-stimulated RA synovial fibroblasts primarily expressed RNA encoding the nonactive form of Xbp, a small amount of RNA encoding the active form was consistently detected (Figure 1b). Further evidence that RA synovial fibroblasts expressed active Xbp1 was obtained by amplification of one of its putative targets, Edem1 [20]. Unlike the results reported for the fibrosarcoma cells [19], CHOP mRNA was not increased in the presence of TNFα. TNFα-stimulated synovial fibroblasts treated with the classical ER stress inducer tunicamycin, however, expressed increased levels of cleaved Xbp1 and CHOP mRNA, confirming that stimulated synovial fibroblasts were capable of eliciting a full ER stress response.

To determine whether RA synovial fibroblasts had a different ER stress response than control fibroblasts we further examined one of the ER stress markers. Phosphorylated eIF2α was present at significantly higher levels in RA synovial fibroblasts stimulated with TNFα compared with nonstimulated RA synovial fibroblasts (*P *< 0.05; Figure 1c). Dermal and osteoarthritis synovial fibroblasts did not show a significant induction of phosphorylated eIF2α upon TNFα stimulation (*P *= 0.17).

These results indicated that TNFα potentiated the acute (protective) ER stress response in RA synovial fibroblasts and that nonstimulated synovial cells grown out from the synovium are undergoing ER stress. This suggests the possibility that *in vivo *activation of synovial fibroblasts results in an acute ER stress response.

### TNFα stimulates macroautophagy

In the previous section, we showed that the three arms leading to the UPR are induced to varying degrees by TNFα. The enhanced state of acute ER stress response in TNFα-stimulated RA synovial fibroblasts suggested that TNFα may influence protein degradation pathways. Depending on the cell type and mode of induction of ER stress, both the ubiquitin/proteasome pathway and the lysosome/autophagy pathway are stimulated in response to ER stress [5].

**Figure 2:**
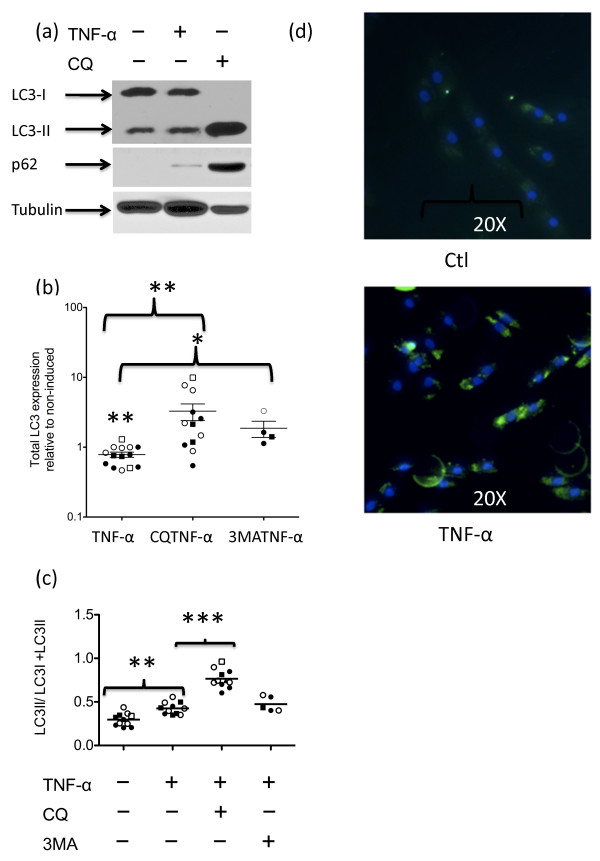
**TNFα affects autophagy**. Lysates from fibroblasts stimulated or not with 10 ng/ml TNFα, 12.5 μM chloroquine (CQ) or 4 mM 3-methyladenine (3-MA) for 24 or 72 hours were analyzed by immunoblotting. **(a) **A representative immunoblot probed with antibodies to microtubule-associated protein 1 light chain 3 (LC3), p62 or tubulin. Results are representative of at least three different rheumatoid arthritis (RA) lines. **(b) **The amount of total LC3 was determined from quantification of LC3-I and LC3-II. Protein loading was corrected by quantification of tubulin. The ratio of total LC3 in TNFα-stimulated cells is compared with their nonstimulated controls. Filled circles, RA lines at 24 hours; open circles, RA at 72 hours; filled squares, control at 24 hours; open squares, control at 72 hours. Significant differences between TNFα-stimulated cultures compared with either non-induced cultures or with cultures that included 3-MA or CQ in addition to TNFα: *P < 0.05, **P < 0.01. **(c) **The ratio of LC3-II to total LC3 was determined by quantification of the band intensities using ImageJ software. Filled circles, RA synovial fibroblasts at 24 hours; open circles, RA synovial fibroblasts at 72 hours; filled squares, control fibroblasts at 24 hours; open squares, control fibroblasts at 72 hours. Significant differences between TNFα-stimulated cultures compared with either non-induced cultures or with cultures that included CQ in addition to TNFα: *P < 0.05, **P < 0.01, ***P < 0.001. **(d) **Fibroblasts grown on chamber slides for 24 hours with or without 10 ng/ml TNFα were examined by fluorescence microscopy for LC3. Nuclei were visualized with DAPI. Results are representative of three lines. Ctl, control.

To determine whether TNFα affected autophagy in fibroblasts, we analyzed the macroautophagy marker LC3 by SDS-PAGE and immunoblotting. During the early stages of autophagy, the LC3 cytosolic form (LC3-I) is conjugated with phosphatidylethanolamine, resulting in the faster migrating LC3-II form [21]. As LC3-II itself is degraded by autophagy, a block in late-stage autophagy will result in its accumulation [22]. p62/SQSTM1 is another marker used for determining autophagic flux [23]. It contains multiple binding domains, including those for ubiquitinated proteins and LC3, and is involved in targeting proteins for degradation. p62 becomes incorporated into autophagosomes as they are forming and is degraded during autophagy. Figure 2a is a representative western blot illustrating that both LC3 forms and p62 were present in RA synovial fibroblasts.

We determined the total amount of LC3 in response to the various treatments. LC3 levels in TNFα-stimulated cells were decreased compared with nonstimulated cells (*P *< 0.01). In contrast, LC3 levels were increased when the autophagy inhibitors chloroquine, a compound that blocks autophagy completion by interfering with the function of lysosomes [11] (*P *< 0.01), or 3-MA, a compound that blocks macroautophagy (*P *< 0.05), were included with TNFα (Figure 2b). There was a statistically significant increase in the amount of the LC3-II macroautophagy indicator band relative to the total LC3 when cells were cultured with TNFα over an extended period of time (*P *= 0.001). This autophagy-stimulating effect of TNFα occurred in all fibroblast lines tested (controls as well as RA). When chloroquine was included in addition to TNFα, a further increase in LC3-II relative to total LC3 levels was observed (*P *< 0.001). However, no significant difference was observed when the macroautophagy inhibitor 3-MA was included with TNFα compared with TNFα alone (*P *= 0.37; Figure 2c). This is probably because 3-MA functions upstream of autophagosome formation. The fact that there was a further accumulation of LC3-II in the presence of chloroquine indicated that TNFα stimulated LC3 processing [21]. In agreement with this, qualitative immunofluorescence staining revealed increased LC3 staining with TNFα (Figure 2d). Together, the data suggest that macroautophagy is induced by TNFα.

### Inhibition of autophagy in the presence of TNFα results in proteasome activation in RA synovial fibroblasts

**Figure 3:**
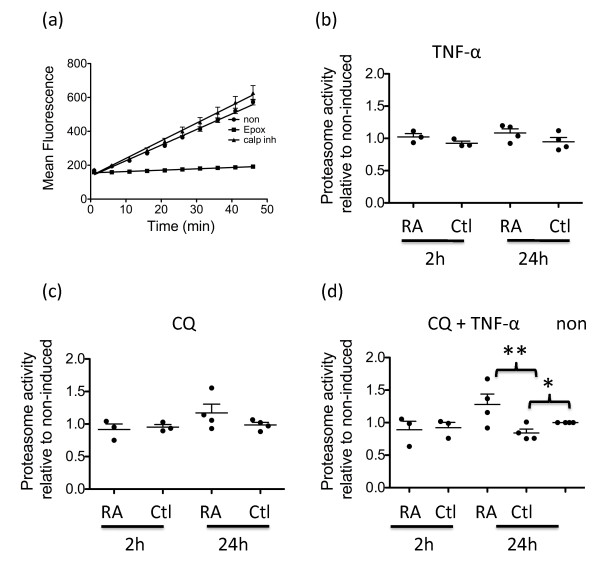
**Proteasome activity is increased by TNFα stimulation and lysosome inhibition**. **(a) **Validation of the chymotrypsin activity assay. Fibroblasts were cultured in EBSS for 2 hours with the addition of 0.5 μM epoximicin or 50 μM calpain inhibitor XI. The substrate Suc-Leu-Leu-Val-Tyr-AMC and digitonin were then added to the wells and fluorescence was measured every 5 minutes for a 45-minute period. **(b) **Fibroblasts were cultured with or without 10 ng/ml TNFα for 2 or 24 hours before substrate addition. Slopes were determined and relative proteasome activity is represented as the slope of TNFα-induced cells relative to their non-induced controls. Ctl, control; RA, rheumatoid arthritis. **(c) **Fibroblasts were cultured with 12.5 μM chloroquine (CQ) for 2 or 24 hours prior to addition of the substrate. Results are presented as the ratio of the activity in the presence of CQ compared with the absence of CQ. **(d) **Fibroblasts were cultured for 1 hour with 12.5 μM CQ before the addition of 10 ng/ml TNFα for 2 or 24 hours. Results are presented as the ratio of the activity in the presence of CQ plus TNFα compared with that in the presence of TNFα. Significant differences in TNFα-stimulated compared with non-induced cultures of control cells or significant differences between the response of control cultures compared with the response of RA synovial fibroblast cultures: *P < 0.05, **P < 0.01.

To directly test whether the proteasome was activated by TNFα, a cell-based assay was used to measure activity of the proteasome [15]. The substrate used in this assay, LLVY, is a substrate for both the chymotrypsin-like proteasome activity [24] as well as calpain activity [25]. It was therefore necessary to determine the specificity for this substrate. The specific proteasome inhibitor epoxomicin inhibited the activity by 97 to 98% while the cell-permeable calpain inhibitor XI slightly increased the activity (Figure 3a). This observation confirmed that the majority of the activity measured by this assay was attributable to the proteasome. Proteasome activity measurements were performed at 2 hours to check for a direct effect of inhibitors on the proteasome and at 24 hours, a time point used for other assays in this study. Our results revealed that in some RA synovial fibroblasts, proteasome activity was increased in the presence of TNFα (Figure 3b). However, this increase was not statistically significant.

We confirmed that the assay did not measure proteolysis resulting from autophagy by including the autophagy inhibitor chloroquine (Figure 3c). Surprisingly, when chloroquine was included in addition to TNFα, control and RA fibroblasts responded differently (*P *= 0.01). A further increase in proteasome activity was observed in some RA synovial fibroblasts while a significant decrease in proteasome activity was observed in all control fibroblasts compared with non-induced cells (*P *< 0.05; Figure 3d). This observation indicates that TNFα does not significantly increase proteasome activity directly. When autophagy is blocked, however, proteasome activity increases in RA synovial fibroblasts, possibly as a compensation mechanism.

### RA synovial fibroblasts exhibit increased proteolysis of long-lived proteins when autophagy is blocked in the presence of TNFα

Our results suggested that TNFα induced LC3 processing in all fibroblasts in a manner consistent with autophagy upregulation, yet had little effect on proteasome activity. To confirm these observations, we examined the influence of TNFα on the flux of long-lived proteins [23]. Proteins degraded by autophagy are typically long-lived while those degraded by the proteasome are short-lived [26]. In preliminary experiments, we included inhibitors of the proteasome, autophagy or both to determine the source of the counts. These experiments revealed that the proteolysis measured by this technique could be partly inhibited by a proteasome inhibitor in addition to chloroquine, suggesting our assay measured degradation of long-lived proteins occurring through either the autophagy or proteasome pathways.

**Figure 4:**
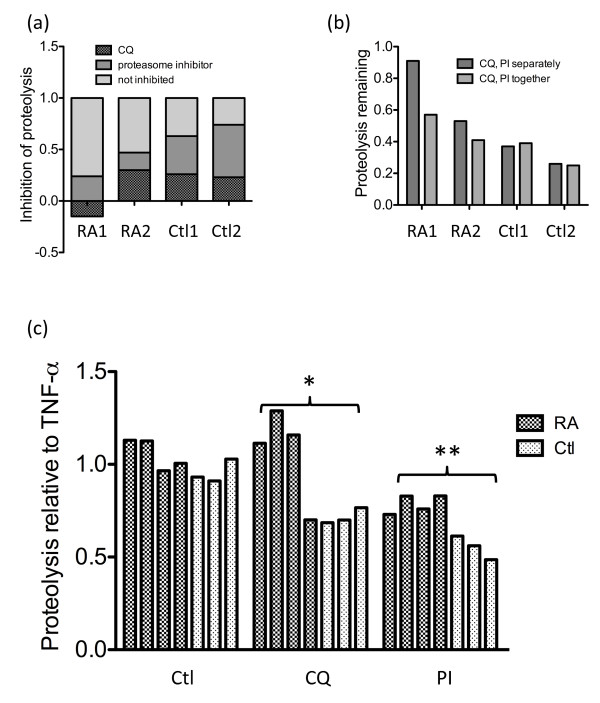
**Rheumatoid arthritis synovial fibroblasts exhibit increased proteolysis of long-lived proteins when autophagy isblocked in the presence of TNF-α**. **(a) **The source of the counts in the long-lived protein degradation assay was determined by comparing the protein flux of long-lived proteins for two rheumatoid arthritis (RA) lines and two control (Ctl) lines cultured for 24 hours with 10 ng/ml TNFα with that of cells cultured with 12.5 μM chloroquine (CQ) or 0.5 μM proteasome inhibitor (PI) in addition to 10 ng/ml TNFα. **(b) **The effect of the protein degradation pathways on each other in RA synovial fibroblasts and control fibroblasts was determined by comparing the proteolysis remaining after the addition of 12.5 μM CQ or 0.5 μM PI to separate wells or the same well of the same cell line for 24 hours. **(c) **The proteolysis remaining after cells were cultured without TNFα or with12.5 μM CQ or 0.5 μM PI in addition to 10 ng/ml TNFα was compared with that when cells were cultured with 10 ng/ml TNFα for 24 hours. This was determined for four RA lines and three control lines. Significant differences in control lines compared with RA synovial fibroblast lines: *P < 0.05, **P < 0.01.

Interestingly, the majority of the proteolysis in the RA lines could not be inhibited by either inhibitor alone (Figure 4a). We therefore examined the possibility that the autophagy and proteasome protein degradation pathways influenced each other. This was accomplished by comparing the proteolysis remaining when the inhibitors were added separately with that when they were added together. In control cells, the remaining proteolysis was the same regardless of whether the inhibitors were added separately or together. In contrast, RA lines had less proteolysis remaining when the inhibitors were added together compared with when they were added separately (Figure 4b). This observation suggested that the two protein degradation pathways functioned independently of each other in control cells whereas they influenced each other in RA synovial fibroblasts.

As shown in the compiled results of four different RA synovial fibroblast lines and three different control fibroblast lines (Figure 4c), TNFα by itself had minimal effect on the degradative flux of long-lived proteins. RA synovial fibroblasts had significantly more proteolysis remaining compared with control fibroblasts following either lysosome inhibition with chloroquine (*P *< 0.05) or proteasome inhibition with a proteasome inhibitor (*P *< 0.01). This factor suggested that RA synovial fibroblasts were better able to compensate for the inhibition of either protein degradation pathway than control fibroblasts. This compensation may be relevant to the survival of RA synovial fibroblasts.

### Ubiquitinated proteins accumulate following proteasome or lysosome inhibition

**Figure 5:**
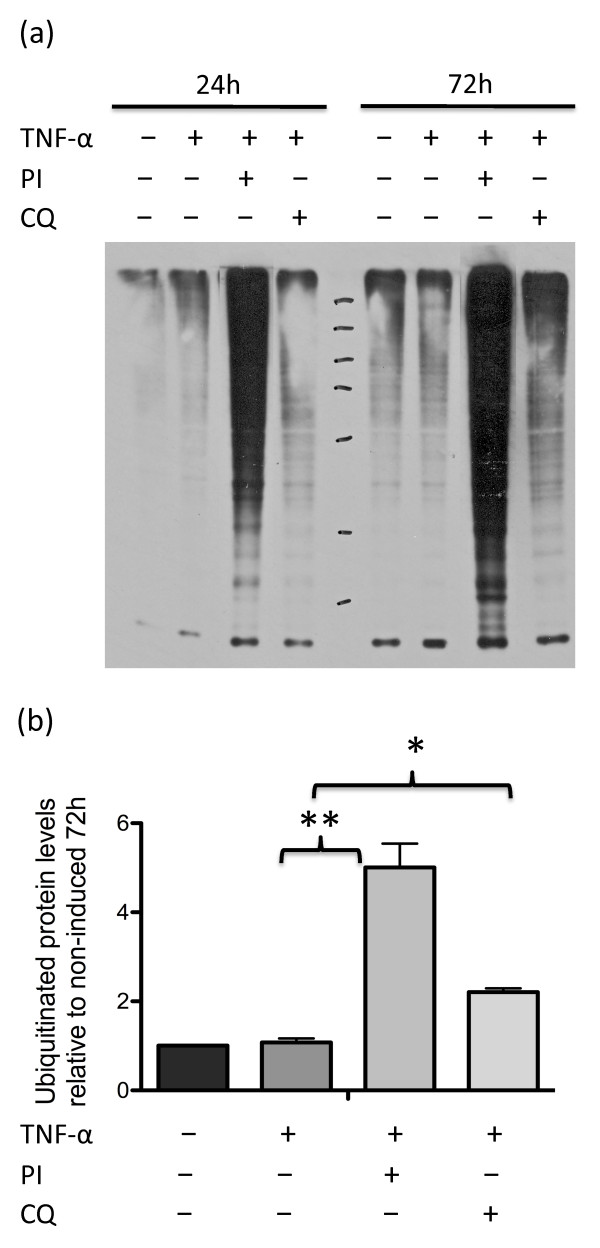
**Ubiquitinated proteins build-up in rheumatoid arthritis synovial fibroblasts with inhibited proteasome or autophagy pathways**. **(a) **Lysates (3 μg) from synovial fibroblasts cultured as indicated with 10 ng/ml TNFα, 0.5 μM proteasome inhibitor (PI) or 12.5 μM chloroquine (CQ) for 24 or 72 hours were immunoblotted and probed for ubiquitinated proteins. **(b) **Quantification of the blots was determined by ImageJ software. These results are the mean and standard deviation of three different experiments. Significant differences in TNFα-stimulated cultures compared with cultures where PI or the autophagy inhibitor CQ was included in addition to TNFα: *P < 0.05, **P < 0.01.

Although ubiquitinated proteins are considered to be primarily degraded by proteasomes, there is increasing evidence that they are also degraded by autophagy [27,28]. We assessed the presence of ubiquitinated proteins in RA synovial fibroblasts by western blot analysis as a complimentary measure of protein degradative pathway activity. TNFα had no effect on the accumulation of ubiquitinated proteins. Inhibition of proteasome activity in the presence of TNFα, however, resulted in a time-dependent (24-hour to 72-hour) build-up of ubiquitinated proteins (Figure 5a). Relative amounts of ubiquitinated proteins in cells cultured with protein degradation pathway inhibitors compared with TNFα-treated cells at 72 hours are shown in Figure 5b. Although inhibition of the proteasome resulted in a greater build-up of ubiquitinated proteins (*P *< 0.01), a significant build-up was also observed when autophagy was inhibited in the presence of TNFα (*P *< 0.05) - suggesting both protein degradation pathways are utilized in the clearance of ubiquitinated proteins. The fact that the banding patterns of proteins on the gel appears to be similar in proteasome-inhibited or lysosome-inhibited (chloroquine) cells suggests that many ubiquitinated proteins may be degraded by either pathway.

### Lysosome or proteasome inhibition affects expression of endoplasmic reticulum stress markers

**Figure 6:**
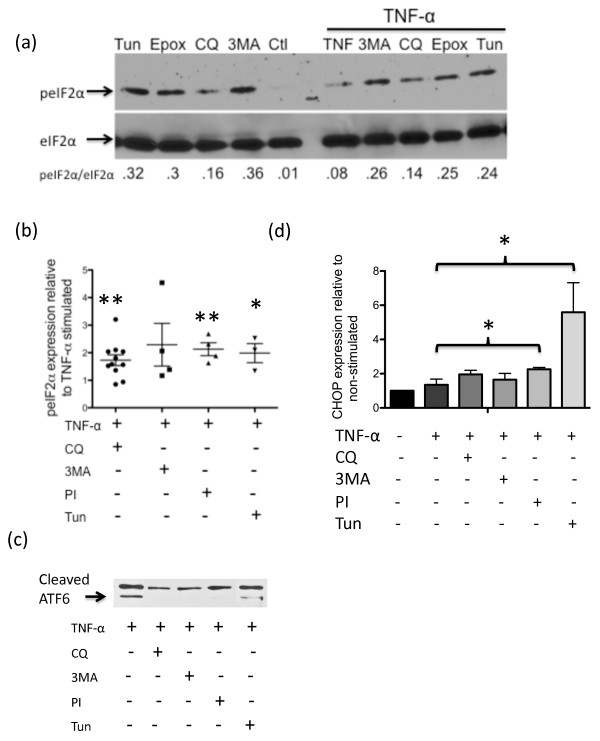
**Lysosome or proteasome inhibition results in endoplasmic reticulum stress**. **(a) **Rheumatoid arthritis (RA) synovial fibroblasts were cultured with 2 μg/ml tunicamycin, 0.5 μM epoxomicin, 12.5 μM chloroquine (CQ) or 10 mM 3-methyladenine (3-MA) for 1 hour prior to the addition or not of 10 ng/ml TNFα for 24 hours. A representative blot probed for expression of phosphorylated eukaryotic initiation factor 2 alpha (peIF2α) or eukaryotic initiation factor 2 alpha (eIF2α) is shown. Blots were scanned and analyzed by ImageJ software. **(b) **The relative amount of phosphorylated eIF2α in cells stimulated with 12.5 μM CQ, 10 mM 3-MA, 0.5 μM epoxomicin or 2 μg/ml tunicmycin in addition to TNFα was determined using tubulin as a loading control. These were then compared with the peIF2α levels in TNFα-stimulated control cells. Significant differences in TNFα-stimulated cultures compared with cultures where the autophagy inhibitors CQ or 3-MAaproteasome inhibitor (PI) or tunicamycin were included in addition to TNFα: *P < 0.05, **P < 0.01. **(c) **The effect of 12.5 μM CQ, 10 mM 3-MA, 0.5 μM epoxomicin or 2 μg/ml tunicmycin in addition to TNFα on the expression of the cleaved/active form of ATF6 was determined. Blot shown is representative of at least three different lines. **(d) **CHOP mRNA levels were determined in cells that had been stimulated for 24 hours with the inhibitors as indicated. They were compared with nonstimulated cells. The mean and standard deviation of CHOP expression in three different lines is shown. Significant differences in TNFα-stimulated cultures compared with cultures where PI or tunicamycin was included in addition to TNFα: *P < 0.05.

Cells use ubiquitination as a method for targeting unwanted proteins for degradation. We therefore queried whether the build-up of ubiquitinated proteins observed following inhibition of the proteasome or lysosome impacted the ER stress response of the RA synovial fibroblasts. To address this question, we prepared cellular lysates from fibroblasts treated with TNFα and the various inhibitors for 24 hours and then examined them by immunoblotting for the ER stress indicator proteins phosphorylated eIF2α and cleaved ATF6. A typical blot for phosphorylated eIF2α expression is shown in Figure 6a. In the absence of TNFα, all of the treatments were associated with at least a 16-fold increase in the phosphorylated form of eIF2α compared with eIF2α. In the presence of TNFα, however, the phosphorylated eIF2α to eIF2α ratio was already increased and there was only an additional threefold further increase upon treatment with the known ER stress inducer tunicamycin or inhibition of autophagy or proteasome. At 72 hours there was significantly increased phosphorylated eIF2α expression when cells were cultured with chloroquine (*P *< 0.01), epoxomicin (*P *< 0.01) or tunicamycin (*P *= 0.05) in addition to TNFα (Figure 6b). Surprisingly, the amount of cleaved/active ATF6 decreased as early as 24 hours after inhibition of either the proteasome or autophagy (Figure 6c). We detected spliced (active) versions of Xbp1 mRNA upon amplification (data not shown). CHOP expression was significantly increased when cells were cultured in TNFα in the presence of proteasome inhibitor or tunicamycin (*P *< 0.05). Together these results suggest that both the proteasome and autophagy protein degradation pathways influence the ER stress response of RA synovial fibroblasts.

### Proteasome inhibition in the presence of TNFα affects expression of autophagy markers

**Figure 7:**
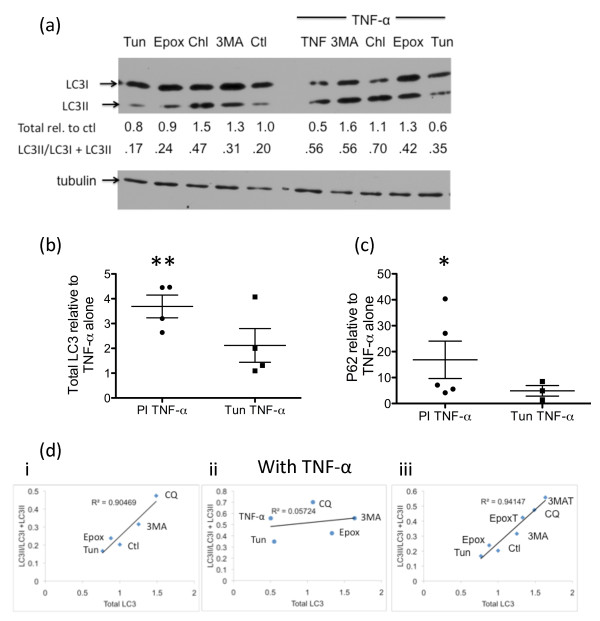
**Proteasome inhibition affects expression of autophagy markers**. **(a) **Rheumatoid arthritis (RA) synovial fibroblasts were cultured with 2 μg/ml tunicamycin, 0.5 μM epoxomicin, 12.5 μM chloroquine (CQ) or 10 mM 3-methyladenine (3-MA) for 1 hour prior to the addition or not of 10 ng/ml TNFα for 24 hours. Cellular lysates were immunoblotted and probed for microtubule-associated protein 1 light chain 3 (LC3) or tubulin as a loading control. Band intensities were quantified using ImageJ software. **(b) **The total amount of LC3 in the samples relative to that in the TNFα sample was determined. Significant differences in TNFα-stimulated cultures compared with cultures where a proteasome inhibitor was included in addition to TNFα: **P < 0.01. **(c) **The total amount of p62 in the samples relative to that in the TNFα sample was determined. Significant differences in TNFα-stimulated cultures compared with cultures where a proteasome inhibitor was included in addition to TNFα: *P < 0.05. **(d) **LC3 results from (a) are plotted to show the relationship between total LC3 and LC3-II relative to total LC3: (i) without TNFα, (ii) with TNFα, (iii) samples treated with proteasome inhibitor or macroautophagy inhibitor in the presence of TNFα shown in (ii) replotted with the samples without TNFα treatment shown in (i) to show the restoration of the linear relationship between total LC3 and LC3-II relative to total LC3. This suggests that the decreased LC3 levels observed in the presence of TNFα result from proteasome activity as well as macroautophagy. In all cases, results are representative of at least three different experiments.

In the absence of TNFα, total LC3 levels were decreased by culture with the known ER stressor tunicamycin or epoxomicin and, as expected, were increased with the autophagy inhibitors 3-MA or chloroquine (Figure 7a). In contrast, in the presence of TNFα, total LC3 levels were significantly increased with the autophagy inhibitors (Figure 2b) or proteasome inhibition (*P *< 0.01; Figure 7b). Expression of p62 was also significantly increased relative to TNFα when the proteasome was inhibited (*P *< 0.05; Figure 7c). As shown in Figure 7d, there was an excellent linear correlation between the amount of LC3 relative to control and the ratio of LC3-II relative to total LC3 in the absence of TNFα. This correlation was lost when TNFα was included but was regained when either the proteasome inhibitor epoxomicin or the macroautophagy inhibitor 3-MA was included, suggesting that the decreased LC3 levels observed in the presence of TNFα were attributable to proteasome activity as well as macroautophagy.

### Increased resistance to proteasome and autophagy/lysosome inhibitors by RA synovial fibroblasts

**Figure 8:**
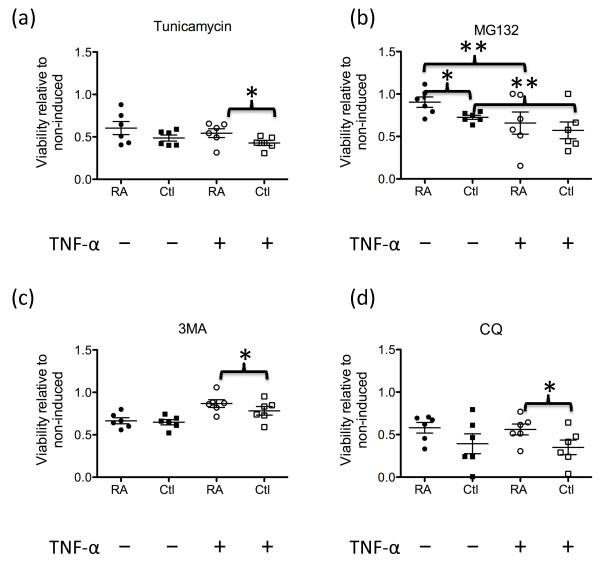
**Rheumatoid arthritis synovial fibroblasts are more resistant to proteasome and autophagy/lysosome inhibitors than other fibroblasts**. Fibroblasts (*n *= 6 rheumatoid arthritis (RA), *n *= 3 osteoarthritis, *n *= 3 dermal), stimulated or not with 10 ng/ml TNFα, were cultured with **(a) **2 μg/ml tunicamycin, **(b) **0.5 μM MG132, **(c) **4 mM 3-methyladenine (3-MA) or **(d) **12.5 μM chloroquine (CQ) for 72 hours. An XTT assay was performed and the survival of the cells was determined. Bars show standard error of the mean. Significant differences in control fibroblasts compared with RA synovial fibroblasts or the response of TNFα-stimulated cultures compared with non-induced cultures: *P < 0.05, **P < 0.01.

In this study we have shown that synovial fibroblasts use both the autophagy and proteasome degradation pathways. To determine the biological significance of these pathways for fibroblast viability, we treated the cells for 72 hours with the proteasome inhibitor MG132 (0.5 μM), the ER stress inducer tunicamycin (2 μg/ml), the lysosome inhibitor chloroquine (12.5 μM), or the macroautophagy inhibitor 3-MA (4 mM) in the presence or absence of TNFα, and then assessed their viability by an XTT assay. As it had been reported that RA synovial fibroblasts were more resistant to ER stress inducers than osteoarthritis synovial fibroblasts, we included three osteoarthritis synovial fibroblast lines and three skin fibroblast lines in our experiments as controls. An XTT assay determined that there was no difference in TNFα sensitivity between the RA synovial fibroblasts and the control fibroblast lines (*P *= 0.43). TNFα-stimulated RA synovial fibroblasts cultured with the known ER stress inducer tunicamycin were significantly more viable than similarly treated control cells (*P *< 0.05; Figure 8a). Decreased viability occurred with MG132, chloroquine and 3-MA, confirming that both the proteasome and lysosome degradation pathways were used by fibroblasts to maintain their viability.

Interestingly, unstimulated RA synovial fibroblasts were relatively resistant to the proteasome inhibitor MG132 and there was a significant difference between the viability of control fibroblasts compared with RA synovial fibroblasts (*P *= 0.01; Figure 8b). This suggested that an alternative protein degradation system such as the lysosome/autophagy pathway was sufficient to maintain viability of RA synovial fibroblasts in the absence of TNFα. In the presence of TNFα, however, MG132 was significantly more effective at decreasing cell viability in all fibroblasts (*P *< 0.01) - suggesting that, under these conditions, the proteasome degradation pathway was required to maintain fibroblast viability.

In the presence of TNFα, RA synovial fibroblasts were more resistant than control cells to the macroautophagy inhibitor 3-MA (*P *< 0.05; Figure 8c) or the lysosome inhibitor chloroquine (*P *< 0.05; Figure 8d). In long-lived protein degradation assays, the contribution of macroautophagy to the total autophagy can be approximated as the percentage of protein degradation inhibitable by lysosome inhibitors that is also inhibitable by the macroautophagy inhibitor 3-MA [29]. We therefore used this approach to determine the contribution of macroautophagy to cell survival. The contribution of macroautophagy to the total autophagy was greater in RA synovial fibroblasts than in the control fibroblasts (78.5% vs. 57%) in the absence of TNFα. In the presence of TNFα, the contribution of macroautophagy to total autophagy declined to 32% in RA synovial fibroblasts and to 34% in control fibroblasts. This revealed that macroautophagy was the most important autophagy pathway in RA synovial fibroblasts in the absence of TNFα (78.5%). To rule out the possibility that the decreased cellular viability after chloroquine treatment was due to lysosome rupture resulting in the release of cathepsins into the cytosol, we treated the cells with a cathepsin inhibitor and observed that this failed to rescue the cell viability (data not shown). This indicated that intralysosomal cathepsins were contributing to synovial fibroblast survival rather than causing cellular necrosis.

Together, the results from this set of experiments suggested that macroautophagy played an important contribution to the viability of RA synovial fibroblasts in the absence of TNFα while proteasomes were important for the viability of RA synovial fibroblasts in the presence of TNFα. These results also suggest that, compared with other fibroblasts, RA synovial fibroblasts have more active proteasomal and lysosomal pathways.

## Discussion

In this study, we examined the effect of TNFα on the ER stress response and protein degradation pathways in RA synovial fibroblasts to determine whether these are potential mechanisms enabling the increased survival of synovial fibroblasts in RA.

We assessed the expression of molecules within each of the UPR signaling pathways to determine whether the pathways were activated. Following 72 hours of culture with TNFα, we observed increased expression of phosphorylated eIF2α and the active form of ATF6 relative to nonstimulated RA synovial fibroblasts. We also observed a small amount of the spliced Xbp1 mRNA but our experiments were not designed to determine whether this was increased compared with nonstimulated cells. CHOP expression was not significantly altered with TNFα stimulation. Together, our results suggest that fibroblasts are under acute ER stress and that adjustments in the UPR signaling pathways in the presence of TNFα are made to enable continued quality control of the proteins passing through the ER.

Ubiquitin/proteasome and lysosome/autophagy are two main pathways used by cells to eliminate proteins causing ER stress. Given that TNFα is a key cytokine driver in RA synovium, our aim was to determine whether TNFα influenced either of these protein degradation pathways. TNFα substantially modified LC3 expression, as evidenced by a decrease in total LC3 levels and an increase in the membrane-associated LC3 form in all fibroblasts. Our findings are supported by the recent observation of the effect of TNFα on LC3 processing in Ewing sarcoma cells [30], MCF-7 cells [31] and human skeletal muscle cells [32]. When chloroquine, a lysosome inhibitor, was added with TNFα, the levels of the lower LC3 band were further increased. p62 expression was in agreement with LC3 expression. Since TNFα stimulated LC3 processing and turnover, these results suggested that TNFα modulated the autophagy pathway. As the cells in our experiments were cultured under normal conditions with full serum, the TNFα-modulated autophagy pathway is unlikely to be the typical autophagy pathway activated under starvation conditions and probably represents a constitutive pathway.

To clarify the significance of autophagy-associated protein modulation in TNFα-stimulated fibroblasts, we determined the flux of long-lived proteins, generally considered to represent autophagy flux. To determine what this assay was measuring in our system, we initially inhibited the autophagy and proteasome degradation pathways separately. This inhibition revealed that the assay measured degradation occurring through both pathways as well as through a mechanism that we have not yet identified. When we included chloroquine and a proteasome inhibitor separately or together in dermal fibroblasts, their effect was additive - suggesting that the pathways proceeded independently of each other. In RA synovial fibroblasts, however, the effect of the inhibitors was not additive, suggesting that the protein degradation pathways influenced each other. RA synovial fibroblasts were significantly more resistant than control fibroblasts to the inhibition of protein flux through either the autophagy pathway or the proteasome degradation pathway. Together, these results suggest that the protein degradation pathways in RA synovial fibroblasts influence and compensate for each other.

We employed a chymotrypsin-like activity assay to gain further evidence that the proteasome was activated in response to TNFα or chloroquine. We observed that three of the four RA synovial fibroblast lines cultured with TNFα or chloroquine for 24 hours had increased chymotrypsin-like activity compared with those cultured without TNFα. In contrast, three of four control lines examined had decreased chymotrypsin-like activity compared with those cultured without TNFα. This suggested that, in RA synovial fibroblasts, TNFα is not only capable of inducing expression of E3 ubiquitin ligases involved in the ubiquitination pathway [33] but may also stimulate the proteasome itself. This hypothesis is in agreement with the long-lived protein degradation assay that suggested RA synovial fibroblasts, but not control fibroblasts, attempt to compensate for lysosome inhibition by activating the proteasome. To date, most studies examining the increased activity of the ubiquitin/proteasome pathway have concentrated on the regulation of the ubiquitination of proteins. A few studies, however, have demonstrated that the proteasome itself can be regulated [34-36]. Presently we do not know how TNFα or lysosome inhibition stimulates proteasome activity in RA synovial fibroblasts.

There are a number of examples where the autophagy pathway is activated to compensate for proteasome inhibition [37]. This may in fact happen in the RA synovial fibroblasts cultured in the absence of TNFα as they are relatively insensitive to proteasome inhibition. In contrast to our studies, a reduction of proteasomal activity in cell lysates prepared from neuroblastoma SHSY5Y cells [38] and SK-N-SH cells [39] treated with chloroquine has been reported. This was attributed to a proteasome inhibitory affect of chloroquine. According to our results, however, the proteasome in RA synovial fibroblasts can be induced to degrade long-lived proteins if autophagy is inhibited. This is the first example of which we are aware where proteasome activation occurs in response to autophagy inhibition. This suggests that the proteasome and autophagy interface is deregulated in RA synovial fibroblasts.

Treatment of fibroblasts with inhibitors of the two main protein degradation pathways revealed that both pathways contributed to fibroblast survival. TNFα stimulated autophagy in all fibroblast lines and caused a shift in the usage of the lysosome/autophagy pathways from primarily 3-MA sensitive to more chloroquine sensitive, suggestive of a switch from macroautophagy to chaperone-mediated autophagy. This is supported by studies of mouse embryo fibroblasts that also were shown to undergo a decrease in macroautophagy upon TNFα stimulation [29]. In the absence of TNFα, fibroblasts from patients with RA were significantly more resistant to proteasome inhibition than control fibroblasts. In contrast, TNFα-stimulated fibroblasts required an active ubiquitin/proteasome pathway for survival and TNFα-stimulated synovial fibroblasts from patients with RA were significantly more resistant to inhibition of the lysosome/autophagy pathway and tunicamycin-induced ER stress than other fibroblasts. We conclude that constitutive lysosome/autophagy is more active in unstimulated RA synovial fibroblasts compared with control fibroblasts while ubiquitin/proteasome pathways are more active in TNFα-stimulated RA synovial fibroblasts, possibly enabling them to better tolerate ER stress than non-RA fibroblasts. Unstimulated fibroblasts appear to survive with a functional lysosome/autophagy pathway while TNFα stimulation necessitates a functional proteasomal pathway.

There are a number of potential explanations for proteasome requirement in the presence of TNFα. For example, TNFα not only stimulates cytokine expression but also results in accumulation of reactive oxygen species that may damage proteins. Both of these scenarios may necessitate the removal of additional aberrant or excess proteins. Furthermore, the classical method for NF-κB activation requires that its inhibitor, IκB, be degraded by the proteasome [40]. As TNFα activates NF-κB, which in turn activates transcription of prosurvival molecules, inhibition of the proteasome would result in inhibition of NF-κB and a change in the balance of prosurvival molecules to proapoptotic molecules. In some diseases, such as Alzheimer's disease and inflammatory bowel disease, there is evidence that ER stress can lead to an inflammatory response that is linked to their pathogenesis [41,42]. The inflammatory response serves to alert neighboring cells of the impending stress to prevent further tissue damage. This has been suggested to occur through ER stress-induced pathways such as PERK-eIF2α that activate the NF-κB signaling pathway, the main pathway leading to inflammatory responses. As RA is an inflammatory disease associated with activated NF-κB, the fibroblast-associated ER stress possibly contributes to the initiation and inflammation associated with the pathology of the disease. Interestingly, proteasome inhibitors have been shown to be effective in relieving inflammation in the rat models of bacterial cell-wall-induced polyarthritis [43] and adjuvant-induced arthritis [44].

Although hydroxychloroquine has been used for many years in the treatment of RA, the base is slow acting and how the treatment functions in controlling the disease is unclear. The bioavailability in patients with RA is between 0.22 and 0.83 μM [45], considerably below the 12.5 μM chloroquine used in this study. Interestingly, clinically relevant doses of chloroquine also inhibit lysosomal function, although at a slower rate and suboptimally [46]. This suggests that hydroxychloroquine may be functioning in RA patients by partially inhibiting autophagy, required for synovial fibroblast viability.

There is a report that LC3 may be degraded by proteasome processing [47]. Our results support this report as we observed increased LC3 levels following proteasome inhibition and decreased levels when the proteasome was activated with TNFα. Additionally, the percentage of the lower form was increased in the presence of TNFα. As the lower form is membrane associated while the upper form is cytoplasmic, possibly only the upper form is available for degradation by the proteasome and thus the apparent shift in LC3-I to LC3-II occurs depending on the activity of the proteasome. Similarly, although p62 was originally reported to be specifically degraded by autophagy [48], this marker has also been shown to increase when the proteasome is inhibited [39]. If LC3 and p62 are degraded by the proteasome, the macroautophagy pathway would no longer be available and could explain the shift from the usage of macroautophagy to other forms of autophagy and proteasome-mediated protein degradation observed after TNFα stimulation in this study and the mouse embryo fibroblast study [29].

## Conclusions

Our findings suggest that fibroblasts are under continuous ER stress that is increased by TNFα. The fibroblasts use both the proteasome and autophagy pathways to clear aberrant proteins and promote cell survival. Compared with control fibroblasts, non-induced RA synovial fibroblasts have more macroautophagy and are more resistant to proteasome inhibition, suggesting that they have more active lysosome/autophagy pathways enabling them to compensate for proteasome inhibition. TNFα stimulates autophagy in RA synovial fibroblasts, and there appears to be a switch from primarily macroautophagy usage to other forms of autophagy and dependence on a functional proteasome. If completion of autophagy is blocked, RA synovial fibroblasts are uniquely able to compensate for the inhibition by upregulating the proteasome, suggesting the proteasome and autophagy interaction is deregulated in RA synovial fibroblasts. This suggests that therapeutically targeting both arms of the protein degradation pathways may be of benefit in diseases such as RA that are associated with an increased tolerance to ER stress.

## Abbreviations

ATF: activation of transcription factor; Bip/Grp78: immunoglobulin binding protein/78-kDa glucose regulated protein; BSA: bovine serum albumin; eIF2α: eukaryotic initiation factor 2 alpha; ER: endoplasmic reticulum; IRE1α: inositol-requiring transmembrane kinase and endonuclease 1 alpha; LC3: microtubule-associated protein 1 light chain 3; 3-MA: 3-methyladenine; NF: nuclear factor; PBS: phosphate-buffered saline; PCR: polymerase chain reaction; PERK: protein kinase-like endoplasmic reticulum kinase; RA: rheumatoid arthritis; TCA: trichloroacetic acid; TNF: tumor necrosis factor; UPR: unfolded protein response; Xbp-1: X-box-binding protein-1.

## Competing interests

The authors declare that they have no competing interests.

## Authors' contributions

AMC participated in the study design, writing of the manuscript, data analysis and performed the experiments. NM and RG participated in the study design and acquisition of tissue samples. ECK and SAB participated in the study design, writing of the manuscript and data analysis. All authors read and approved the manuscript.
